# From predicted bacteriocins to ecological function

**DOI:** 10.1016/j.crmicr.2026.100662

**Published:** 2026-09-10

**Authors:** Samson Baranzan Wayah, Kensuke Arakawa, Koshy Philip

**Affiliations:** a Graduate School of Environmental, Life, Natural Science, and Technology, Okayama University, Okayama, Japan; b Department of Biochemistry, Faculty of Life Sciences, Kaduna State University, Kaduna, Nigeria; c Institute of Ocean and Earth Sciences, University of Malaya, Kuala Lumpur, Malaysia; d Faculty of Medicine, Nursing and Health Sciences, SEGi University, Selangor, Malaysia

**Keywords:** Antimicrobial gene prediction, Bacteriocins, Context-dependent interactions, Ecological function, Genome mining, Microbial ecology

## Abstract

Genome and metagenome mining have uncovered large repertoires of predicted bacteriocin loci, but ecological interpretation has lagged behind discovery. This review uses bacteriocins as a focused model for a broader problem in antimicrobial gene prediction: sequence identifies encoded potential, but it does not establish expression, product deployment, target engagement, or community-level consequence. We distinguish four forms of bacteriocin “silence”: transcriptional silence, where loci are not detectably expressed under tested conditions; conditional silence, where expression or activity emerges only under specific environmental or social cues; phenotypic silence, where expression or product formation does not yield detectable activity in routine assays; and ecological silence, where activity is observed but its consequences for coexistence, exclusion, colonization, or community assembly remain unresolved. We also clarify how this framework extends previous reviews of bacteriocin diversity and microbiome-shaping roles by providing an inference-centered taxonomy that separates encoded potential from demonstrated ecological function. Finally, we propose a function-first roadmap for bacteriocin research, including condition-resolved transcriptomics and proteomics, promoter and cue-dissection experiments, expanded target panels, spatially structured assays, defined consortia and microcosms, producer/non-producer comparisons, and explicit tests of resistance, immunity, and fitness consequences. Treating predicted bacteriocin loci as hypotheses rather than conclusions will improve how the field moves from gene catalogs to causal ecological understanding.

## Highlights

• Predicted bacteriocin loci do not by themselves establish ecological function.
• We distinguish transcriptional, conditional, phenotypic, and ecological silence.
• Sequence data reveal encoded potential, but context determines realized function.
• A function-first framework links loci to expression, targets, and outcomes.
• Bacteriocins provide a model for interpreting hidden antimicrobial repertoires.

## Graphical abstract

**Figure:**
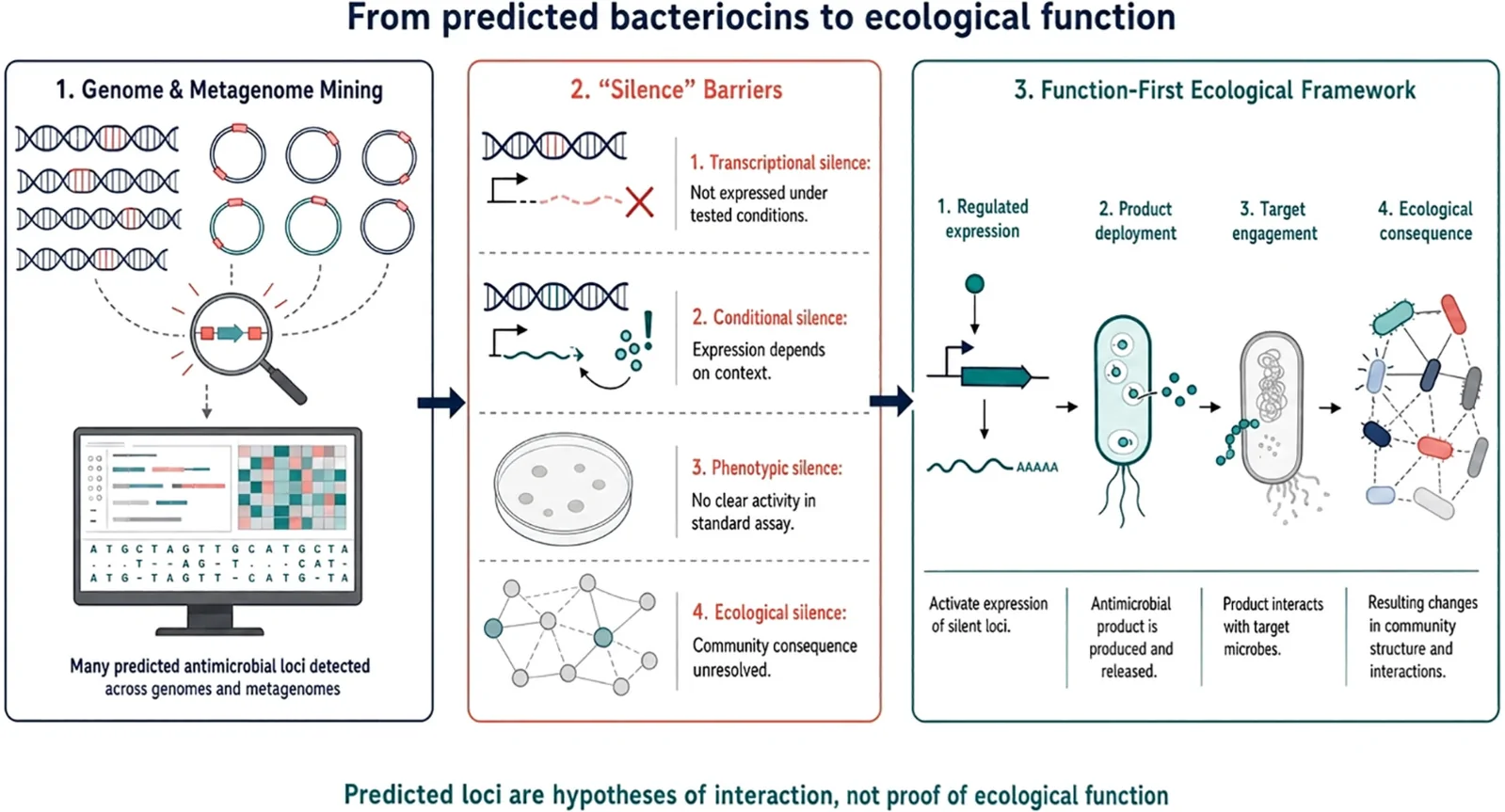

## 1 Introduction

Genome and metagenome sequencing have transformed the search for microbial antagonistic traits. Across isolates, microbiomes, and environmental communities, large-scale mining now reveals vast numbers of loci predicted to encode bacteriocins (Sugrue et al., 2024) and other antimicrobial systems (Hemmerling and Piel, 2022). This progress has shifted the central challenge of the field. The problem is no longer finding candidate genes, but determining when they are expressed, how their products are deployed, which organisms they affect, and whether they matter in natural communities.

This review is deliberately focused on bacteriocins rather than all antimicrobial systems. Bacteriocins provide a tractable model because their genomic architectures are often recognizable, their activities can in principle be tested experimentally, and their ecological effects are strongly context dependent (Wayah et al., 2026). They therefore provide a tractable model for asking how far sequence-based prediction can take us, and where additional evidence is required. Although the same problem applies to other predicted antimicrobial traits, the argument developed here is anchored in the bacteriocin literature.

In bacteriocin research, this gap has created a recurring interpretive error, prediction is often treated as explanation. The existence of a putative bacteriocin locus is often seen as evidence of bacteriocin production potential (Zhang et al., 2025), competitive strategy (Backman et al., 2024), or ecological function (Heilbronner et al., 2021). But a sequence merely shows encoded potential. Between locus detection and ecological consequence lie multiple layers of contingency, including regulation, processing, export, target susceptibility, spatial encounter, and environmental context (Garcia-Gutierrez et al., 2019; Hols et al., 2019). A locus that appears silent in standard laboratory conditions may be important in the structured, nutrient-limited, multispecies settings in which microbes actually live (Chikindas et al., 2018; Granato et al., 2019; Netzker et al., 2018). Conversely, a locus that yields measurable inhibition *in vitro* may have little consequence *in situ* (Dicks et al., 2018; Hibbing et al., 2010).

Two recent reviews provide important foundations for this discussion. Sugrue et al. (2024) synthesized bacteriocin diversity, function, discovery, and application, while Heilbronner et al. (2021) reviewed the microbiome-shaping roles of bacteriocins (Heilbronner et al., 2021; Sugrue et al., 2024). The present review builds on those contributions but has a different aim. Rather than re-reviewing bacteriocin diversity or cataloging known microbiome effects, it asks how strongly different kinds of evidence support claims about ecological function. Its main contribution is an inference framework that separates predicted loci, expression, product deployment, target engagement, and ecological consequence, together with a taxonomy of bacteriocin “silence” that identifies where interpretation fails.

This inference problem is especially clear in bacteriocin research. Their widespread distribution (Choi et al., 2023) and recognizable genetic architectures (Zhang et al., 2023) have made them prime targets for genome mining (Cui et al., 2021; Donia and Fischbach, 2015), comparative genomics (Santarelli et al., 2025; Sun et al., 2025), and metagenomic surveys (Leng and de la Fuente-Nunez, 2025; Wang et al., 2026). As a result, the number of predicted bacteriocin systems now far exceeds the number that have been functionally characterized (Dillen et al., 2026), and functional characterization still outpaces ecological validation (Arbulu and Kjos, 2024). This is not simply a temporary annotation gap. It reflects a deeper disconnect between identifying a putative antimicrobial system and understanding its role in microbial life. Inhibition of an indicator strain is not equivalent to ecological function, nor does it explain whether a molecule contributes to colonization, interference competition, niche defense, or community assembly.

Here, we argue that the central bottleneck in bacteriocin biology is no longer discovery but interpretation. By using bacteriocins as an anchor system, this review examines why predicted antimicrobial genes remain functionally opaque, what kinds of “silence” separate sequence from ecological meaning, and how function-first, context-aware approaches can close the gap between gene discovery and ecological understanding.

### 1.1 Note on review scope and literature identification

This article is a conceptual narrative review rather than a systematic review or meta-analysis. Literature was identified through targeted searches of PubMed, Web of Science, Google Scholar, and publisher databases using combinations of terms including bacteriocin, bacteriocin gene cluster, genome mining, metagenome mining, silent gene cluster, cryptic biosynthetic gene cluster, microbial competition, microbiome, ecological function, antimicrobial activity, quorum sensing, biofilm, spatial structure, immunity, resistance, and community assembly. Priority was given to recent reviews, mechanistic bacteriocin studies, genome and metagenome mining papers, and experimental studies linking bacteriocins to expression, activity, target engagement, or ecological outcome. Because the aim is framework development rather than effect-size estimation, no formal PRISMA-style screening, risk-of-bias assessment, or quantitative synthesis was performed. The resulting literature base should therefore be read as representative rather than exhaustive.

## 2 The many meanings of silence

The term “silent” is often used loosely for predicted antimicrobial loci that remain poorly understood (Hoskisson and Seipke, 2020; Machado et al., 2017), but that usage obscures an important biological distinction. In current practice, silence can refer to a gene cluster that is not transcribed under laboratory conditions (Okada and Seyedsayamdost, 2017), a product that is expressed but inactive in routine assays (Hoskisson and Seipke, 2020; Medema and Fischbach, 2015), or a locus that is bioinformatically recognizable yet still unlinked to any validated phenotype (Hooft et al., 2020). These cases are not equivalent. They represent different barriers between encoded potential and ecological interpretation. If silence is to be analytically useful, it must be resolved into distinct mechanistic and inferential states (Fig. 2).

A practical distinction between transcriptional and conditional silence is especially important. Transcriptional silence should be reserved for cases in which a locus remains undetectable at the RNA level under the conditions tested (Dong et al., 2025). Conditional silence should be used when the same locus is inactive in a baseline condition but becomes expressed, deployed, or phenotypically active after a defined cue or ecological context is supplied (Miller et al., 2018). The distinction therefore depends less on regulatory mechanism, which may overlap, than on experimental evidence: negative expression under one condition *versus* cue-dependent activation across a condition series.

Transcriptional silence describes a predicted bacteriocin locus that is not detectably expressed under tested conditions. This is the most familiar meaning of silence in genome mining and natural product discovery. In this kind of silence, loci are typically referred to as cryptic, as their transcripts or products cannot be detected in standard assays (Covington et al., 2021; Hoskisson and Seipke, 2020). However, lack of detectable expression does not imply lack of biological relevance (Mao et al., 2018; Xu et al., 2019). For instance, the genes encoding Ruminococcin C (RumC) were highly expressed *in vivo* when the producer strain colonized rats but the corresponding transcripts were below the detection level when the strain was grown in a liquid culture medium (Pujol et al., 2011). Also, Li and O’Sullivan (2002) demonstrates that the transconjugant *Enterococcus* sp. strain N12β, which carries the entire nisin operon, initially shows a lack of nisA transcription via Northern hybridization and reverse transcriptase polymerase chain reaction when grown under standard conditions (Li and O’Sullivan, 2002). Bacteriocin loci are often regulated by narrow and environmentally sensitive programs. These include quorum sensing (Valente et al., 2026; Wyllie and Jensen, 2025), nutrient limitation (Abbasiliasi et al., 2017; Kuo et al., 2013), stress (El-Sharoud et al., 2022; Kang et al., 2022), host-derived signals (Gillor et al., 2008; Hertzog et al., 2018), or competitor-dependent cues (Majeed et al., 2011; McAnulty et al., 2024). A transcriptionally silent locus therefore marks not biological absence, but failure to identify the conditions that trigger expression.

Conditional silence describes systems that are functional only under particular environmental, host-associated, spatial, or social conditions. Conditional silence is experimentally resolved by testing a defined matrix of cues, such as cell density, nutrient limitation, host factors, stress, biofilm growth, or competitor identity, and showing that expression or activity appears only in particular contexts. For example, expression of the streptococcin B bacteriocin locus scbABC is tied to the early competence cascade in *Streptococcus pneumoniae* through a ComE binding site in the promoter. It shows how activation can depend on a specific physiological state (Richardson et al., 2025). They also mentioned that the expression of the scb locus, which encodes streptococcin B, can be activated independently of the competence pathway. This occur only when driven by an exogenously supplied peptide cue from *Streptococcus pseudopneumoniae*, a related competitor (Richardson et al., 2025). Another study shows that the *Streptococcus pneumoniae* Blp bacteriocin locus is expressed in a quorum-sensing-dependent manner. Consequently, mediating intra-strain competition and turnover within the host. In addition, the Blp locus can be induced by competence stimulating peptide (Aggarwal et al., 2024).

Phenotypic silence is when a bacteriocin locus is transcribed and its product processed or released, but no evident impact is shown in a conventional test (Harrington et al., 2021; Wang et al., 2018). This may be due to testing the wrong target, the substance being active at short range or low concentration, the necessary growth stage being missing, or the assay not capturing ecologically important effects (Anderssen et al., 1998; Dinglasan et al., 2025). Phenotypic silence therefore cautions against treating “no activity detected” as evidence of no function. To buttress this point, Lagos et al. (2001) identifies mutants in genes *mceC, mceI*, and *mceJ* that secrete an inactive form of microcin E492 which is still able to form ion channels in a phospholipidic bilayer, despite the lack of observed activity in a standard cell lawn assay. Additionally, they reported that the inactive microcin isolated from mutants in *mceC* and *mceJ* does have a lethal effect when incubated with spheroplasts of sensitive cells, suggesting a defect in outer membrane receptor recognition rather than an inability to function as a channel-former (Lagos et al., 2001). A different study determines that transparent variants of *Streptococcus pneumoniae* produce equivalent amounts of the blpMNPO transcript as opaque variants when stimulated with a synthetic pheromone, but transparent variants exhibit no pneumocin MN-mediated inhibitory activity in a standard overlay assay (Dawid et al., 2009).

Ecological silence occurs when activity is measurable at the molecular or organismal level, but its consequences for coexistence, exclusion, colonization, or community assembly remain unresolved (Riley and Wertz, 2002). A bacteriocin may inhibit a susceptible strain *in vitro*, yet whether it shapes natural communities depends on whether producers and targets co-occur, whether they encounter one another at relevant distances, whether resistance or immunity blunts the effect, and whether local inhibition scales into a durable ecological outcome (Dobson et al., 2012). For instance, a study demonstrated that the lacticin 3147-producing strain, *Lactococcus lactis* DPC6520, while capable of surviving transit through the gastrointestinal tract, lacked antimicrobial efficacy against *Clostridium difficile* and *Listeria monocytogenes* in the models of infection used (Dobson et al., 2011). In another investigation, it was observed that lacticin 3147 and pediocin PA-1 displayed synergy in anti-*Clostridioides difficile* activity during agar-based screening. Consequently, confirming measurable bacteriocin activity. However, the combined bacteriocins unexpectedly increased *Clostridioides difficile* levels in a Simplified Human Intestinal Microbiome (SIHUMI-C) consortium, despite having synergy in pure culture assays (Ríos Colombo et al., 2025).

These forms of silence are conceptually distinct but often overlap in practice. A locus that appears transcriptionally silent in one setting may prove conditionally expressed in another. A system that is clearly expressed may remain phenotypically silent because the relevant target has not been tested. A robust inhibition phenotype may still be ecologically silent if its effects disappear in multispecies or spatially structured settings. The value of distinguishing these categories is sharper inference: each category points to a different missing experiment and a different level of evidence needed to connect sequence to ecological function.

## 3 From locus to ecological role: why sequence alone is not enough

A predicted bacteriocin locus is an important clue, but it is only the beginning of biological inference. Sequence can identify a putative system, suggest aspects of its architecture, and sometimes indicate likely maturation, transport, or immunity functions (Sugrue et al., 2024; van Belkum et al., 2011). What it cannot do on its own is determine whether that system is expressed under relevant conditions, whether its product is successfully deployed, whether susceptible targets are encountered, or whether any resulting interaction alters ecological outcomes. Between locus detection and ecological role lies a series of biological filters, each of which can preserve, reshape, or eliminate the functional significance of encoded potential (Fig. 1).

**Fig. 1:**
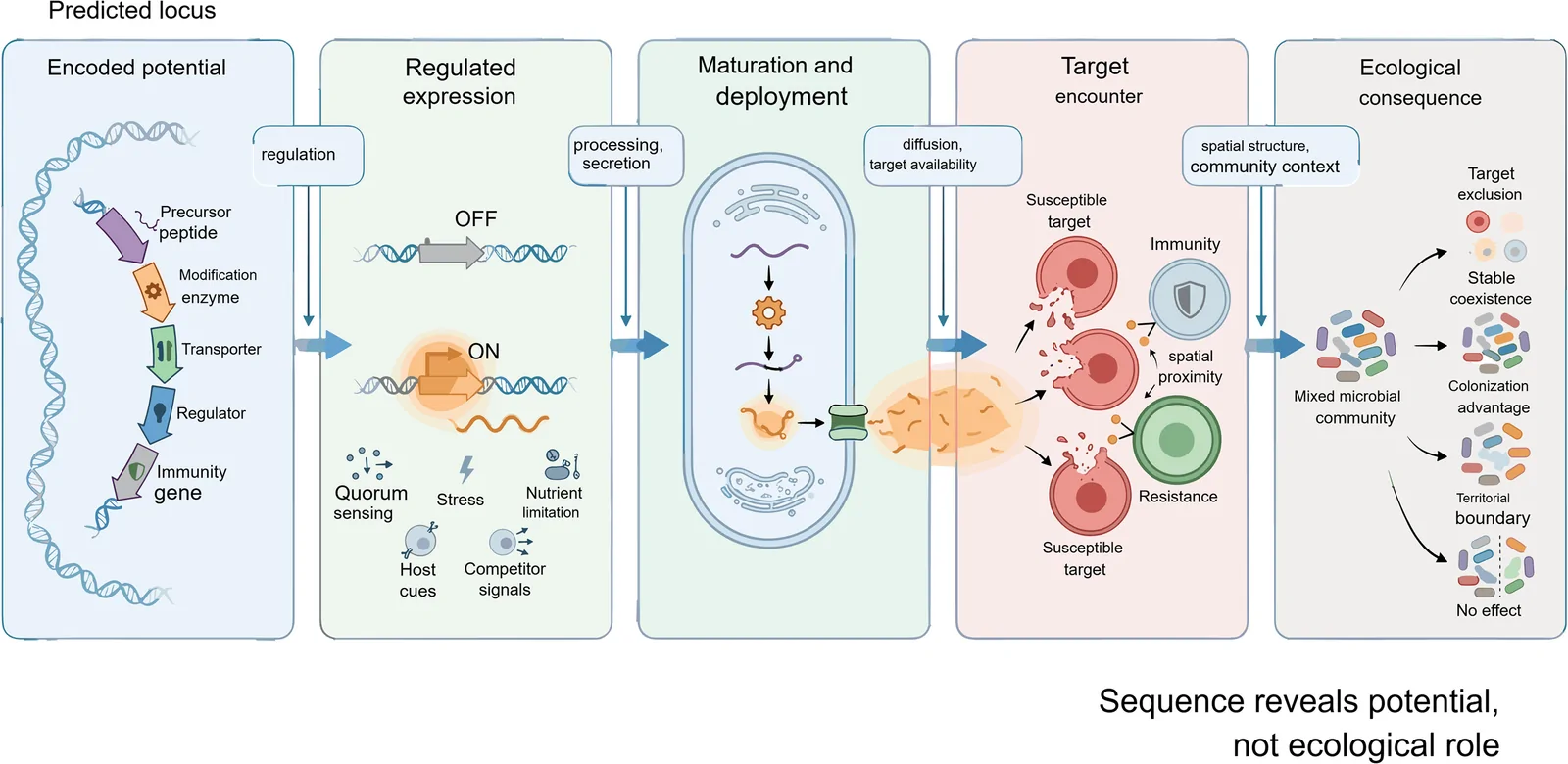
From predicted locus to ecological consequence. Sequence-based discovery can identify putative bacteriocin loci and reveal encoded antimicrobial potential, but ecological function emerges only after passage through multiple biological filters. These include regulated expression, maturation and deployment of the product, encounter with susceptible targets, and translation of local antagonism into community-level consequence. At each step, inference may fail because expression is conditional, deployment is spatially constrained, susceptibility is unevenly distributed, or ecological outcomes depend on community context. Bacteriocin function should therefore be understood not as a direct property of sequence alone, but as the outcome of context-dependent transitions linking genomic potential to ecological effect.

**Fig. 2:**
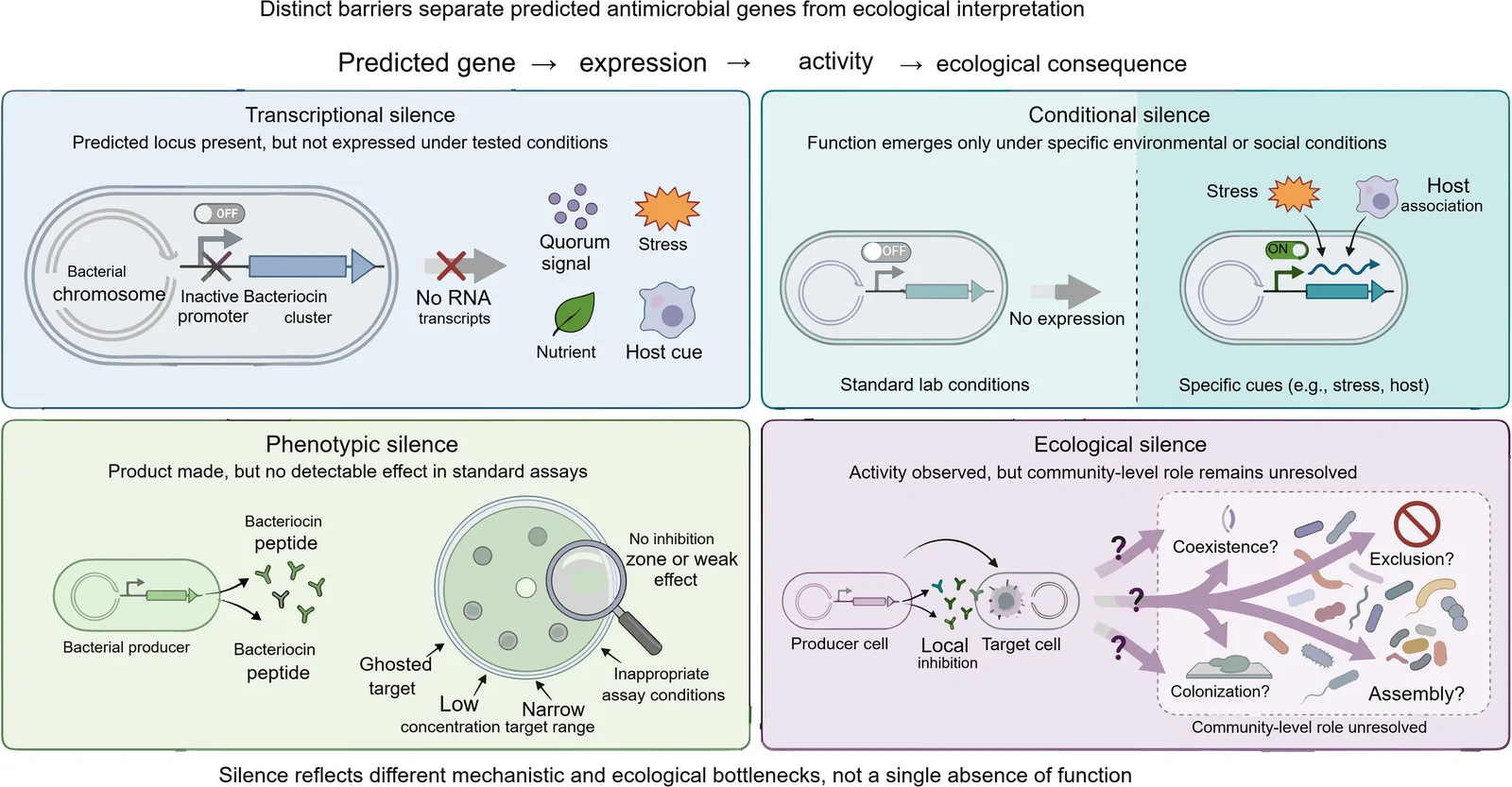
The many meanings of silence. "Silence" in bacteriocin biology does not describe a single absence of function, but multiple points at which inference can fail between predicted genes and ecological interpretation. Transcriptional silence refers to loci that are present but not detectably expressed under tested conditions. Conditional silence describes systems that are activated only under specific environmental, spatial, or social cues and therefore remain invisible in generalized assays. Phenotypic silence applies when a locus is expressed and may produce a mature product, yet no clear activity is detected in routine laboratory screens, often because the relevant targets or conditions are missing. Ecological silence refers to cases in which activity can be observed, but its consequences for coexistence, exclusion, colonization, or community assembly remain unresolved. Distinguishing these forms of silence clarifies that the gap between prediction and function reflects multiple mechanistic and ecological bottlenecks rather than a single class of uncharacterized loci.

Available validation studies illustrate the size of this inferential gap, even if a comprehensive field-wide validation rate is not yet available. For example, Zhang et al. (2025) synthesized 26 hypothetical bacteriocins from gut commensal species and found antibacterial activity for 16. This reveals that a substantial fraction of computationally predicted candidates did not show activity in that experimental context (Zhang et al., 2025). Rosenkilde et al. (2024) similarly showed that inhibitory patterns among heterologously expressed class II bacteriocins changed across *in vitro, ex vivo*, and *in vivo* complexity (Rosenkilde et al., 2024). These examples do not provide a universal validation rate, but they support the more cautious claim that prediction-to-function conversion is incomplete and context dependent.

The first level is encoded potential. Genome mining works well because many bacteriocin loci contain recognizable features, including precursor peptides, modification enzymes, transport components, regulators, and immunity determinants. These signatures make bacteriocins unusually tractable compared with many other cryptic interaction systems (Sugrue et al., 2024). But detectability can create a false sense of understanding. A locus that resembles a known bacteriocin system does not necessarily produce an active molecule, and even when it does, its ecological role may differ from what annotation implies (Nes et al., 2007; Piper et al., 2011).

The next filter is regulated expression. Many bacteriocin loci are not constitutively active, but induced by quorum sensing (Proutière et al., 2021), nutrient limitation (Vermeulen et al., 2021), stress (Kang et al., 2022), host cues (Hertzog et al., 2018), or competitive interactions (Majeed et al., 2011). A locus can therefore remain invisible in standard laboratory conditions while being important in specific ecological settings (Chia et al., 2025; Pujol et al., 2011). Regulation determines not only whether a bacteriocin is produced, but also under what circumstances the costs of production are justified. Sequence alone rarely reveals this logic of activation.

Even when a locus is expressed, function depends on maturation and deployment. Many bacteriocins require post-translational modification, proteolytic processing, secretion, and release into the local environment before they can act (McAuliffe et al., 2001; Perez et al., 2018). In structured communities, products may remain localized near producers (García-Bayona et al., 2017; Meza-Torres et al., 2021), diffuse unevenly (Garcia-Garcera and Rocha, 2020), or be degraded before reaching targets (Fernandes et al., 2024; Mathur et al., 2018). Sequence can sometimes predict machinery, but not actual release, persistence, spread, and concentration *in situ*.

A further filter is target engagement. For a bacteriocin to matter, an appropriate target must be present, susceptible, and encountered under relevant conditions (Bashey, 2015). Susceptibility depends on receptor availability (Diep et al., 2007), physiological state (Patel et al., 2021), immunity, resistance, and local environment (Sugrue et al., 2024). A product with strong activity against an indicator strain *in vitro* may have little ecological relevance if corresponding targets are absent or protected in natural communities.

The final step is ecological consequence. Even when expression, deployment, and target inhibition occur, the outcome for a community may remain uncertain (Ríos Colombo et al., 2023). An antagonistic event does not automatically scale into competitive exclusion, stable coexistence, altered succession, or host-associated persistence. Ecological role is an emergent property of interaction networks, spatial structure, resistance and immunity, and environmental state, not a direct extension of molecular activity.

## 4 Why bacteriocins are the right model system

Bacteriocins are a particularly useful system for examining the gap between predicted genes and ecological function because they combine genomic tractability with ecological contingency. Many bacteriocin loci are readily detected by sequence-based approaches. This is due to the fact that they contain recognizable precursor peptides, modification enzymes, transport machinery, regulatory elements, and immunity determinants (Wayah et al., 2026). At the same time, the presence of these loci says little on its own about when they are expressed, how they are deployed, which organisms they affect, or whether they alter community dynamics.

Their value is reinforced by broad phylogenetic and ecological distribution. Bacteriocins occur across diverse bacterial lineages (Choi et al., 2023) and are associated with host-associated (Gu et al., 2024), terrestrial (Subramanian and Smith, 2015), aquatic (Chen et al., 2024; Nayak et al., 2022), plant-associated (Helfrich et al., 2018; Nazari and Smith, 2020), and food-linked environments (Yi et al., 2022). They therefore do not represent a niche feature of a few specialized taxa, but a recurring component of microbial interaction across habitats.

Bacteriocins are also informative because they sit at the interface of mechanism and ecology. They are mechanistically grounded enough to be traced from sequence, but ecologically contingent enough to show why sequence alone is insufficient. Their roles depend on who produces them, which competitors are present, how susceptibility and immunity are distributed, whether producers and targets encounter one another, and what costs or benefits govern production (Caballero-Flores et al., 2023; Chassaing and Cascales, 2018).

Finally, bacteriocins make evolutionary questions experimentally tractable. Their production can be costly (Inglis et al., 2009), their activity is often targeted (Bali et al., 2016), and their consequences are shaped by immunity and resistance (Kennedy and Comstock, 2024). These features place them within clear frameworks of conflict, kin discrimination, niche defense, and coevolution. They are therefore valuable not only as antimicrobial products, but as a model for how hidden interaction traits are maintained and expressed in microbial communities.

## 5 Ecology is the missing layer

Ecological function is often invoked in discussions of bacteriocins, but much less often demonstrated. Most evidence for bacteriocin activity comes from simplified conditions that show what a molecule can do, not what it does in the structured, heterogeneous, multispecies environments where microbial interactions unfold (Arbulu and Kjos, 2024; Dobson et al., 2012; Yao et al., 2022). Even when loci are detected, expressed, and shown to inhibit a target *in vitro*, their ecological role remains unresolved unless those activities are placed in the contexts that govern encounter, susceptibility, and community response (Fig. 3).

**Fig. 3:**
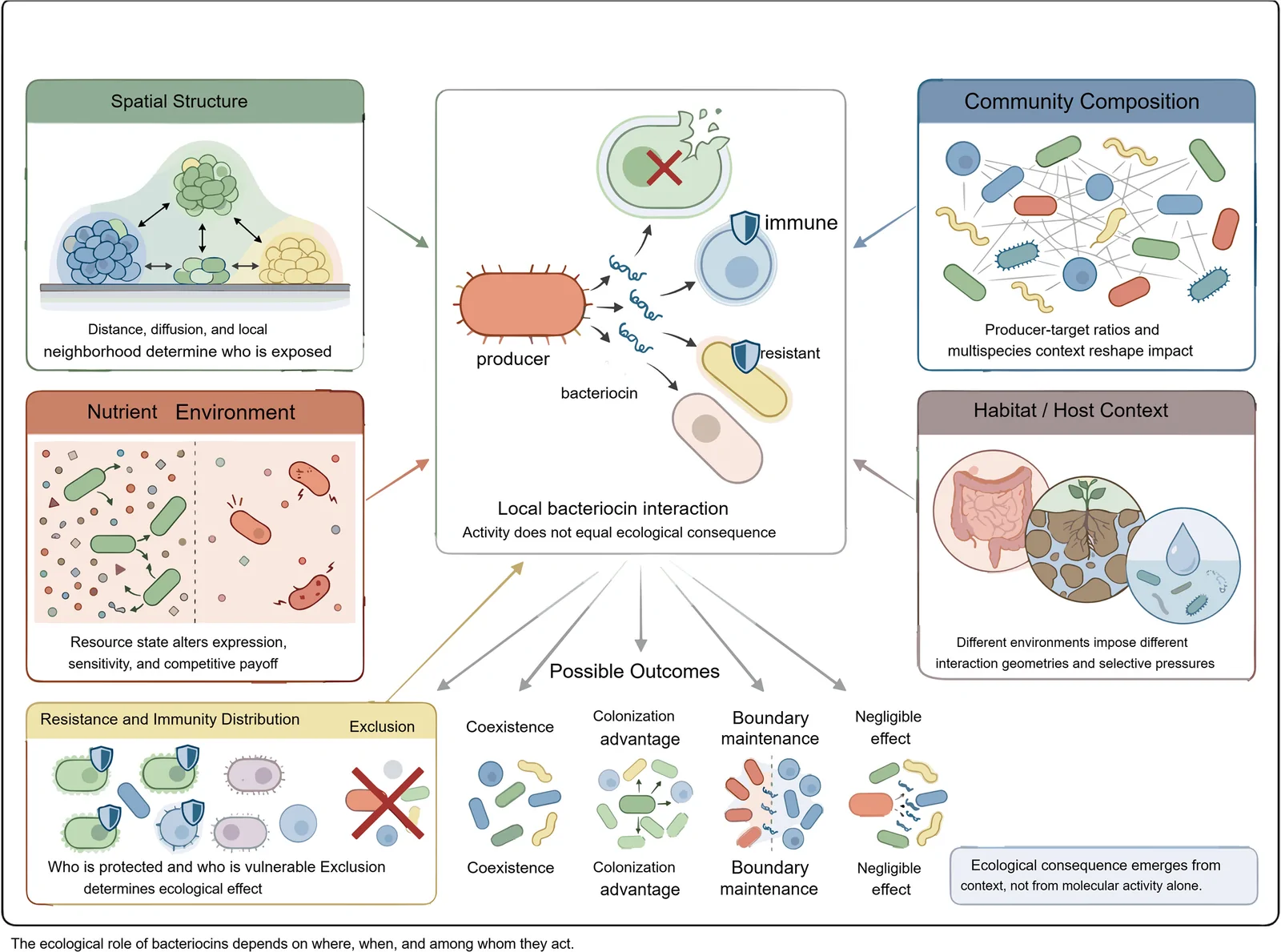
Ecology determines outcome. The ecological effects of bacteriocins are not fixed properties of the molecule alone, but emerge from context. Spatial structure influences diffusion, retention, and local encounter among producers and targets. Community composition determines which susceptible, resistant, or immune populations are present and how direct antagonism propagates through interaction networks. Nutrient environment shapes both bacteriocin production and target susceptibility by altering physiological state, stress, and competitive payoff. Habitat or host context imposes distinct interaction geometries and selective pressures across gut, soil, aquatic, plant-associated, and other microbial settings. The distribution of resistance and immunity further determines who is vulnerable, who is protected, and whether inhibition scales into exclusion, coexistence, colonization advantage, boundary maintenance, or negligible effect. Ecological consequence therefore depends on where, when, and among whom bacteriocins act, rather than on antimicrobial activity alone.

One major determinant of ecological consequence is spatial structure. Microbes often grow in biofilms (Paula et al., 2020), aggregates (Wessel et al., 2014), mucus layers (Martens et al., 2018), particles (Rosato et al., 2020), and other structured microhabitats in which interactions are localized (Batsch et al., 2024; Dar et al., 2021). Under these conditions, bacteriocin effects depend not only on potency, but also on distance (Klein et al., 2020), diffusion (Yanni et al., 2019), retention (Gharsallaoui et al., 2016), and neighborhood composition (Wang et al., 2024). A product that appears weak in bulk culture may be effective in a dense local patch, whereas one that performs well in liquid assays may have little impact if it diffuses poorly or is rapidly neutralized *in situ*.

A second determinant is community composition. Ecological outcome depends on which taxa are present, in what abundance, and in what arrangement relative to the producer (Kong et al., 2018; Majeed et al., 2011). Target range alone is not enough to predict function. The density of susceptible competitors (Almeida-Santos et al., 2021), the presence of partially resistant strains (Gerardin et al., 2016), the abundance of close relatives carrying immunity (Veening and Blokesch, 2017), and indirect effects of removing one taxon from a network (Weiss et al., 2023) can all reshape consequence.

The nutrient environment adds a further layer of contingency. Resource availability (Nie et al., 2023), growth phase (Mataragas et al., 2003), stress (Parlindungan et al., 2019), and disturbance (Cornforth and Foster, 2013) can influence both bacteriocin production and target susceptibility. Producers may invest in antagonism only when competition intensifies under limitation or when exclusion becomes especially valuable. Targets may also change in sensitivity depending on physiological state.

Habitat and host context shape function as well. Gut-associated, soil, marine, plant-associated, and food-linked communities impose different interaction geometries, disturbance regimes, and opportunities for recurrent contact among producers and targets (Li and Rebuffat, 2019; Wayah et al., 2026). These differences affect not only whether bacteriocins are expressed, but what ecological role they are likely to play.

A final determinant is the distribution of resistance and immunity. Producers do not act on passive targets. Susceptible populations may carry resistance, close relatives may possess immunity determinants, and these protections may be unevenly distributed across a community (Leisner and Haaber, 2012; Nadell et al., 2016). As a result, the same bacteriocin can generate exclusion in one setting, frequency-dependent coexistence in another, or little detectable effect in a third (Kirkup and Riley, 2004; Sassone-Corsi et al., 2016).

## 6 The bottleneck and a bacteriocin-specific roadmap forward

The central bottleneck in bacteriocin biology is no longer the discovery of candidate loci, but the conversion of prediction into explanation (Cui et al., 2021). Genome mining, comparative genomics, and metagenomic surveys can now identify putative antimicrobial systems at scale, yet the methods used to assign function have not kept pace (Sugrue et al., 2024; Szymczak et al., 2025). The result is a rapidly expanding catalog of predicted bacteriocins without a corresponding ability to determine when they are expressed, how they are deployed, which targets they encounter, or whether they alter ecological outcomes *in situ*.

A function-first framework should treat predicted bacteriocin loci as hypotheses about context-dependent interaction. The practical goal is to test each transition in the sequence-to-function chain: locus integrity, regulated expression, product maturation and deployment, target engagement, community-level outcome, and evolutionary significance (Table 1). The roadmap below is organized as priority-ranked, bacteriocin-specific experimental steps rather than general methodological aspirations.

**Table 1:** From predicted bacteriocin loci to ecological function: what can be inferred, and what remains unknown. The table summarizes the main levels of inference that separate sequence-based bacteriocin prediction from demonstrated ecological function. For each level, it distinguishes what can be directly detected or measured, what is often inferred from that evidence, what remains unresolved, and which experimental approaches are needed to strengthen causal interpretation. The purpose is to make the function-first framework operational by treating predicted bacteriocin loci as hypotheses to be tested across expression, maturation and deployment, target engagement, ecological consequence, and evolutionary significance.

Level of inference	What can be detected or measured	What is often inferred	What remains unknown	What is needed next
Predicted locus / encoded potential	Genome or metagenome contains precursor, modification, transport, regulator, or immunity genes.	Antimicrobial capacity or ecological role.	Expression, locus integrity, product formation, and ecological relevance.	Comparative genomics plus locus validation; tests of intact precursor, immunity, transport, and regulatory modules.
Regulated expression	RNA, promoter activity, or induction signature under defined cues.	The system is activated during competition or stress.	Whether expression produces mature product and whether activation occurs in the native context.	RT-qPCR/RNA-seq, promoter reporters, regulator mutants, and cue matrices including quorum, stress, nutrients, host factors, and competitors.
Maturation and deployment	Processed peptide, secretion, extracellular product, or transporter activity.	Product is available to affect neighbors.	Diffusion, degradation, retention, delivery range, and local concentration *in situ*.	Proteomics, targeted mass spectrometry, secretion assays, transporter/processing mutants, and spatial localization.
Phenotypic activity	Growth inhibition, killing, delayed growth, or altered competition.	The bacteriocin is ecologically relevant.	Whether targets and assay conditions are ecologically appropriate.	Co-isolated target panels, altered media and growth states, structured assays, and readouts beyond inhibition zones.
Target engagement in context	Producer-target co-occurrence, susceptibility, immunity/resistance distribution, and local encounter.	Relevant targets are affected in the native habitat.	Whether encounter occurs at meaningful distances and times.	Spatial mapping, strain-level susceptibility testing, defined consortia, biofilms, microfluidics, and habitat-relevant microcosms.
Ecological consequence	Changes in abundance, invasion, colonization, coexistence, exclusion, boundaries, or succession.	Bacteriocins shape community assembly or persistence.	Causality, durability, indirect effects, and environmental dependence.	Matched producer/non-producer/complemented strains, immunity or resistance variants, community perturbation experiments, and fitness measurements.
Evolutionary significance	Distribution of bacteriocin, immunity, and resistance traits across strains, habitats, or time.	Production is adaptive and maintained by competition or niche defense.	When costs, resistance, immunity, and community context favor production or loss.	Longitudinal sampling, experimental evolution, cost-benefit assays, and competition experiments across spatial and resource regimes.

Priority 1 **verify locus integrity and regulatory capacity**

Before ecological claims are made, predicted loci should be checked for intact precursor, modification, transport, regulatory, and immunity components (Montalbán-López et al., 2017). Comparative genomics should be paired with targeted polymerase chain reaction (PCR), long-read validation where needed, and promoter/regulator inspection. For bacteriocins, this includes testing whether immunity genes are present and co-localized, because immunity often distinguishes a functional antagonistic system from an incomplete or decaying locus.

Priority 2 **define activating cues experimentally.**

Candidate loci should be assayed across conditions that reflect bacteriocin biology. These includes, quorum or competence signals, nutrient limitation, acid or bile stress, oxidative stress, biofilm growth, host-associated cues, and competitor presence (Guinane et al., 2015). Promoter reporters, RT-qPCR, RNA-seq, and regulator knockouts can distinguish transcriptional silence from conditional activation. This directly addresses whether a locus is inactive or merely untested under the relevant conditions.

Priority 3 **confirm product maturation and deployment.**

Expression alone should not be treated as deployment. Proteomics (Lee et al., 2021), targeted mass spectrometry (Lee et al., 2009), secretion assays (Gajic et al., 2003), and analysis of processing or transporter mutants (Liu et al., 2026) should be used to determine whether a mature bacteriocin is produced, released, retained locally, or degraded. Where possible, producer, processing-deficient, transporter-deficient, and immunity-deficient variants should be compared to separate transcription from functional deployment.

Priority 4 **expand phenotypic testing beyond indicator strains.**

Routine spot-on-lawn assays (Das et al., 2022; Zou et al., 2018) should be complemented with ecologically informed target panels that include co-isolated strains, close relatives, resistant or immune variants, and community members from the habitat of interest. Assays should vary medium, pH, nutrient state, growth phase, oxygen, spatial structure, and producer-target ratios. Readouts should include delayed growth (O’Connor et al., 2024), altered competitive timing (Ríos Colombo et al., 2023), local displacement (Majeed et al., 2011), and spatial patterning (Kirkup and Riley, 2004), not only visible inhibition zones.

Priority 5 **test target engagement in spatial and community context.**

Bacteriocin effects should be tested in systems where encounter structure is explicit. Microcolonies, biofilms, agar surfaces, microfluidic devices, droplets, organoid or mucus models, defined consortia, and semi-natural microcosms can reveal whether producers and susceptible targets meet at distances and frequencies that permit activity (Ríos Colombo et al., 2025; Rutter et al., 2024). Spatial mapping of producers, targets, immunity carriers, and resistant strains is essential for linking molecular activity to realized interaction.

Priority 6 **establish ecological consequence with causal perturbations.**

The strongest evidence requires comparing matched producer, non-producer, complemented, immunity/resistance, and deployment-defective strains in community-scale experiments. Outcomes should include abundance, invasion success, colonization persistence, exclusion, coexistence, boundary maintenance, succession, and resilience after disturbance (Arbulu and Kjos, 2024; Riley and Wertz, 2002). These designs distinguish correlation from causality and clarify whether bacteriocin activity produces durable ecological change.

Priority 7 **quantify fitness costs and evolutionary maintenance.**

Bacteriocin production, immunity, and resistance should be evaluated under the same ecological conditions used to infer function. Competition experiments across spatial structures, nutrient regimes, and community compositions can reveal when production is favored, when resistance is selected, and when immunity or cheater dynamics alter long-term maintenance (Gonzalez and Mavridou, 2019; Niehus et al., 2021). This connects ecological role to evolutionary significance.

## 7 Conclusion: beyond the bacteriocinome

Genome and metagenome mining have transformed bacteriocin biology from a problem of discovery into a problem of interpretation. Hidden repertoires of predicted bacteriocin genes are now easier to detect than to understand. For most loci, the central questions remain unresolved: when they are expressed, how their products are deployed, which organisms they affect, and whether they alter ecological outcomes *in situ*.

The central argument of this review is that the “silent” bacteriocinome is not simply a reservoir of uncharacterized molecules awaiting annotation. It is a map of the points at which inference breaks down between encoded potential and ecological consequence. Silence may reflect lack of expression, context-dependent induction, failure of routine phenotypic assays, or uncertainty about whether measurable activity affects community structure at all. Distinguishing among these forms of silence matters because each identifies a different biological bottleneck and a different experimental need.

Bacteriocins are therefore valuable not only because they may contribute to competition, colonization, and niche defense, but because they provide a model for a broader interpretive problem in biology. Across microbial systems, sequencing reveals hidden functional repertoires faster than those repertoires can be linked to causal outcomes. The challenge is no longer simply to catalog what is present, but to determine what matters, under what conditions, and at what scale.

Moving beyond the bacteriocinome does not mean leaving bacteriocins behind. It means using them to develop a more general approach for interpreting predicted bioactive genes in living systems. Progress will depend on linking genome-scale discovery to condition-resolved expression, localized deployment, target engagement, community consequence, and evolutionary significance. If that link can be made more rigorously, bacteriocin research will do more than clarify one class of antimicrobial traits. It will help define how microbial biology moves from gene catalogs to ecological understanding.

## Funding

The author(s) declared that financial support was received for this work and/or its publication. This work was supported by 10.13039/501100001691Japan Society for the Promotion of Science grant 25KF0249.

## CRediT authorship contribution statement

**Samson Baranzan Wayah:** Writing – original draft, review & editing, Visualization, Conceptualization. **Kensuke Arakawa:** Writing – original draft, review & editing, Methodology. **Koshy Philip:** Writing – original draft, review & editing.

## Declaration of competing interest

The authors declare that they have no known competing financial interests or personal relationships that could have appeared to influence the work reported in this paper.
